# Rapid Control of a SARS-CoV-2 B.1.617.2 (Delta) Variant COVID-19 Community Outbreak: The Successful Experience in Pingtung County of Taiwan

**DOI:** 10.3390/ijerph19031421

**Published:** 2022-01-27

**Authors:** Cherng-Gueih Shy, Jian-He Lu, Hui-Chen Lin, Min-Nan Hung, Hsiu-Chun Chang, Meng-Lun Lu, How-Ran Chao, Yao-Shen Chen, Pi-Sheng Wang

**Affiliations:** 1 Public Health Bureau, Pingtung County Government, Pingtung, Pingtung County 900, Taiwan; pthh7362396@mail.ptshb.gov.tw (C.-G.S.); pth0905981587@mail.ptshb.gov.tw (H.-C.C.); pthmenglun@mail.ptshb.gov.tw (M.-L.L.); 2 Department of Radiology, Pingtung Christian Hospital, Pingtung, Pingtung County 900, Taiwan; 3 Emerging Compounds Research Center, General Research Service Center, National Pingtung University of Science and Technology, Neipu, Pingtung County 912, Taiwan; toddherpuma@mail.npust.edu.tw; 4 Kaohsiung-Pingtung Regional Center, Taiwan Centers for Disease Control, Ministry of Health and Welfare, Executive Yuan, Taipei City 10050, Taiwan; lhuichen@cdc.gov.tw (H.-C.L.); mnhung@cdc.gov.tw (M.-N.H.); 5 Department of Environmental Science and Engineering, College of Engineering, National Pingtung University of Science and Technology, Neipu, Pingtung County 912, Taiwan; 6 Institute of Food Safety Management, College of Agriculture, National Pingtung University of Science and Technology, Neipu, Pingtung County 912, Taiwan; 7 School of Dentistry, College of Dental Medicine, Kaohsiung Medical University, Kaohsiung City 807, Taiwan; 8 Department of Administration, Kaohsiung Veterans General Hospital, Kaohsiung 813, Taiwan; yschen@vghks.gov.tw; 9 School of Medicine, National Yang Ming Chiao Tung University, Taipei 112, Taiwan; 10 Hospital and Social Welfare Organizations Administration Commission, Ministry of Health and Welfare, Nangang, Taipei City 11558, Taiwan; hmpswang@mohw.gov.tw

**Keywords:** Severe Acute Respiratory Syndrome Coronavirus 2 (SARS-CoV-2), Delta-variant, rapid control, vaccination, quarantine, PCR fast screening

## Abstract

The Severe Acute Respiratory Syndrome-associated Coronavirus 2 (SARS-CoV-2) was an outbreak in December, 2019 and rapidly spread to the world. All variants of SARS-CoV-2, including the globally and currently dominant Delta variant (Delta-SARS-CoV-2), caused severe disease and mortality. Among all variants, Delta-SARS-CoV-2 had the highest transmissibility, growth rate, and secondary attack rate than other variants except for the new variant of Omicron that still exists with many unknown effects. In Taiwan, the pandemic Delta-SARS-CoV-2 began in Pingtung from 14 June 2021 and ceased at 11 July 2021. Seventeen patients were infected by Delta-SARS-CoV-2 and 1 person died during the Pingtung outbreak. The Public Health Bureau of Pingtung County Government stopped the Delta-SARS-CoV-2 outbreak within 1 month through measures such as epidemic investigation, rapid gene sequencing, rapidly expanding isolation, expanded screening of the Delta-SARS-CoV-2 antigen for people who lived in regional villages, and indirect intervention, including rapid vaccination, short lockdown period, and travel restrictions. Indirect environmental factors, such as low levels of air pollution, tropic weather in the summer season, and rural areas might have accelerated the ability to control the Delta-SARS-CoV-2 spread. This successful experience might be recommended as a successful formula for the unvaccinated or insufficiently vaccinated regions.

## 1. Introduction

The coronavirus disease 2019 (COVID-19) pandemic, caused by the virus of the Severe Acute Respiratory Syndrome-associated Coronavirus 2 (SARS-CoV-2), currently continues to progress globally. Patients with SARS-CoV-2 infection may have several symptoms and signs, such as non-productive cough, fever, fatigue, myalgia, decreased leukocyte counts, headache, abdominal pain, vomiting, and the disease may further develop into pneumonia, acute respiratory syndrome (ARDS), arrhythmia, or shock [1]. The SARS-CoV-2 was further divided into four genera (Alpha-, Beta-, Gamma-, and Delta-) by the International Committee on Taxonomy of Viruses (ICTV) [2]. Recently, the new SARS-CoV-2 variant, B.1.1.529, had been confirmed on 9 November 2021 and named Omicron by World Health Organization (WHO) on 26 November 2021 [3]. Among them, the B.1.617.2 (Delta) variant of SARS-CoV-2, was first identified in India in late 2020 and has been reported with increased transmissibility and potential reduction in neutralization by some monoclonal antibody treatments. For its potentially higher transmission and secondary attack rates than Alpha-, Beta-, and Gamma- variants [4,5,6], the B.1.617.2 variant has become a variant of concern. In the United States, Delta-variant was the predominant SARS-CoV-2 variant and contributed 99% of the COVID-19 cases and accelerated the increase in hospitalization in mid-September 2021 [7]. Furthermore, the COVID-19 vaccines seem to be less effective against the Delta-SARS-CoV-2 [8]. Therefore, early identification and control of Delta-variant dissemination are essential for limiting COVID-19 impact.

In addition to the main recognized transmission routes of COVID-19, which include respiratory droplets (e.g., talking, coughing, and sneezing) and direct contact [9], several reports showed that the hosts were possibly infected by airborne transmission (e.g., PM_2.5_) under certain environmental conditions, such as poor ventilation or indoor air quality (IAQ), crowds, and lack of masks in indoor environments [10,11,12,13].

Since the outbreak of SARS-CoV-2 disease in December, 2019, the global cumulative number of cases is now more than 261 million, and the global deaths caused by COVID-19 is over 5.2 million [14]. Taiwan also entered the community transmission stage of COVID-19 on 11 May 2021 [15]. On 16 July 2021, the Central Epidemic Command Center (CECC), which is activated by Taiwan Centers for Disease Control (TCDC), announced that Delta-SARS-CoV-2 has been detected in 17 imported and 12 domestic cases in Taiwan [16]. The present study traced the investigation and control of the first imported Delta-SARS-CoV-2 case and the resultant community outbreak in southern Taiwan.

## 2. Materials and Methods

The community outbreak of Delta-SARS-CoV-2 was first found in Fangshan (FS) township (population approximately 5136; 17 km^2^) and Fangliao (FL) township (population approximately 23,200; 58 km^2^), Pingtung County, Southern Taiwan between 14 June and 11 July in 2021. The townships of FS and FL are in the tropical zone, located in Pingtung County, which is the most southern part of Taiwan. Seventeen cases of Delta-SARS-CoV-2 were discovered, and one patient died in this Pingtung outbreak.

The Public Health Bureau of Pingtung County Government (PHB/PTH) followed CECC guidelines including social distancing, mask-wearing, proper sanitization, tracing the origin of the infectious source, quarantine of the suspected coronavirus carriers, and avoiding indoor gatherings to prevent COVID-19 spread in Pingtung since the end of January 2020. Since the begining of the Pingtung outbreak of Delta-SARS-CoV-2, PHB/PTH implemented the rigorous steps, including detailed epidemic investigation and casecontact tracing; rapid isolation expansion; a short period of lockdown of the two involved villages, Fenggang (FG) and Shanyu (SY) in FS; massive antigen screening of SARS-CoV-2; rapid determination of SARS-CoV-2 variant examined by genomic sequence; and accelerated promotion of COVID-19 vaccination to restrain the continuous spread of Delta-SARS-CoV-2 in the outbreak areas.

### 2.1. Detailed Epidemiologic Investigation

There is a legal basis for CECC in Taiwan to conduct outbreak investigations to control, prevent, and comprehend the COVID-19 spread, particularly for community spread in the endemic areas. The detailed epidemiologic investigation, such as contact tracing, source confirmation, and the listing of potentially high-risk infected cases, was collected by the outbreak investigation team for each confirmed case following the CECC guidelines [17].

### 2.2. Quarantine Process in Taiwan

All inbound aircraft and ships, including their crew and passengers, were required to stay at a centralized quarantine station for 14 days to prevent cross-border infectious diseases. After release from quarantine, follow-up health checks may be performed. If people have suspected cold symptoms and signs, they must further perform real-time PCR analysis using SARS-CoV-2 specific primers. In addition, passengers entering Taiwan from high-risk countries (e.g., Indonesia) not only stay at a centralized quarantine station for 14 days but are also subject to real-time PCR analysis directly. This quarantine process helps us conduct more precise and faster epidemiological investigations in Taiwan. People who were diagnosed via a rapid test have been placed in a government-designated quarantine center, or in a hospital, and their health is monitored on a case-by-case basis.

### 2.3. Endemic Area Indicators Cases Selection

In order to implement SARS-CoV-2 epidemic prevention, all of the suspicious COVID-19 cases were quarantined at the quarantine accommodation or the group quarantine facilities for people who came in close contact with confirmed cases. The people were selected by epidemic investigation and the real-link system.

### 2.4. Real-Time PCR Analysis and Sequencing

The nasopharyngeal swab samples were collected from clinically suspected COVID-19 infected individuals and were mixed with viral transport medium. PCR methods are not relevant to this report.

### 2.5. Isolation of High-Risk Infected Population by PHB/PTH

Based on the detailed epidemiologic investigation, the possible Delta-SARS-CoV-2 infected hosts were immediately quarantined at home or in the quarantine accommodation after community spread was confirmed. On 26 June, 2021, PHB/PTH listed the high-risk population to implement rapid expanding isolation for 667 persons, who were quarantined in group quarantine facilities. The suspected hosts in home quarantine or quarantine accommodation were also transferred to the group quarantine facilities.

### 2.6. Fast Vaccination in FS Village

Before the Pingtung outbreak (14 June, 2021), only 3.6% of Taiwanese (855,125 persons) had received the first dose of the AstraZeneca (AZ) or Moderna COVID-19 vaccine. The COVID-19 vaccine to be given to individuals followed the guideline of public funded COVID-19 vaccination order under the national COVID-19 vaccination program. The first three priority groups (group 1–3), including healthcare workers (455,000 persons), central and local government epidemic prevention personnel (90,000 persons), and frontline workers with high risk of contact (35,000 persons) were prioritized for vaccination. Only a few persons in group 4 (those required to travel abroad) received the first doses of the COVID-19 vaccine. Many non-vaccinated citizens lived in FS prior to the outbreak. The rapid vaccination program was implemented with the AZ vaccination in the Pingtung Delta-SARS-CoV-2 endemic areas by PHB/PTH after the outbreak, and 1056 residents in FG and SY villages were vaccinated with the first dose.

### 2.7. Short Lockdown in the Epidemic Areas

In order to control the spread of Delta-SARS-CoV-2 in FS, the supermarkets, convenience shops, restaurants, street vendors, and traditional markets in FG and SY villages, which were epidemic villages, were closed for 3 days. PTH encouraged the residents in these two villages to stay at home and the local government provided the food and materials for epidemic prevention. Local restriction of transportation or mobility was implemented for 3 days in these two villages.

### 2.8. Environmental and Meteorological Conditions

The air monitor station and weather station in Hengchun is closest to the townships of FS and FL among the stations in Taiwan. The air quality, including carbon monoxide (CO), ozone (O_3_), nitrogen monoxide (NO), nitrogen dioxide (NO_2_), PM_10_, PM_2.5_, and sulfur dioxide (SO_2_) levels were gathered from 14 June to 11 July in 2021. The meteorological data, including rainfall, humidity, temperature, and insolation duration, of June and July were recorded. Geographic conditions of FS and FL were also considered.

## 3. Results

From 14 June through 11 July, 2021, 17 cases of Delta-SARS-CoV-2 were identified in the Pingtung outbreak. Initially, 2 cases were returning nationals living in Peru, 13 cases in FS town were first transmitted by Peruvian cases, and 2 cases in FL town were infected by neighbors of Peruvian cases in the indoor environment of the FL hospital. The first Delta-SARS-CoV-2-associated death was a 72-year-old Taiwanese farmer with underlying hypertension and diabetes mellitus. The Taiwanese government, specifically PTH, was successful in controlling and preventing Delta-SARS-CoV-2 spread through epidemic investigation, rapid gene sequencing, rapid expanding isolation measures, expanding screening of the COVID-19 antigen for people who lived in regional villages, rapid COVID-19 vaccination, and short regional lockdown and travel restrictions, leading to no new cases of Delta-SARS-CoV-2 infection within the community since 26 June, 2021.

**Figure 1:**
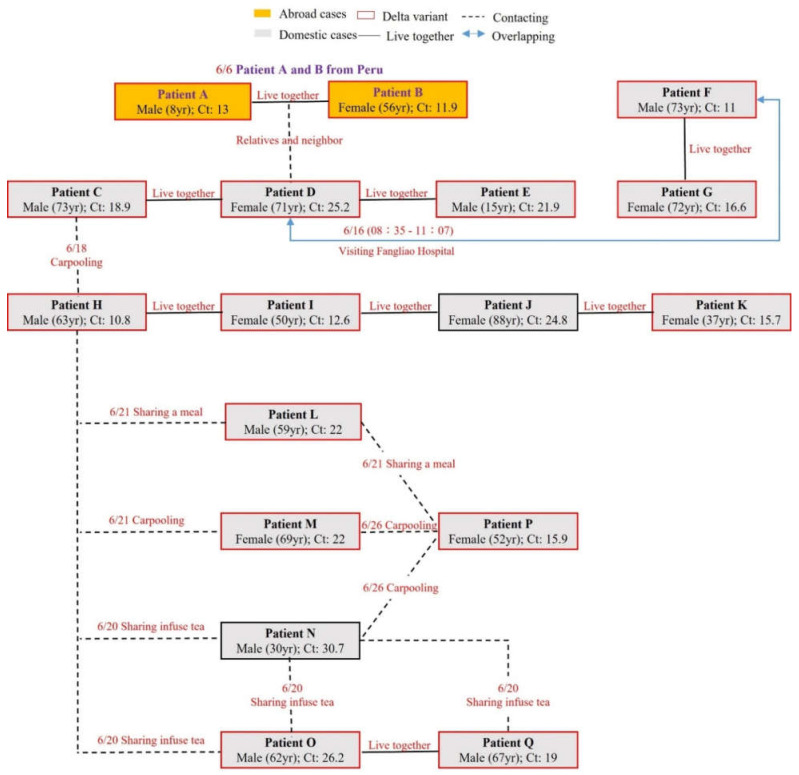
The patient-patient relationship.

The patient-patient relationship is shown in Figure 1. PHB/PTH found the originally infectious source, which was imported by a grandmother (Patient A) and grandson (Patient B) returning from Peru on 6 June, 2021. Their neighboring relative (Patient D) acquired the Delta-SARS-CoV-2 during their quarantine period while taking out the garbage and having a short fleeting conversation with the relative next door. Subsequently, Patient D transmitted the Delta-SARS-CoV-2 to Patient F and G when she visited an outpatient waiting area at an FL Hospital with respiratory symptoms. Patient F was suspected to have caught the Delta-variant from Patient D due to overlapping time in the same hospital and waiting room for hours. Patients C and E lived together in the same household as Patient D. Patient H was a taxi driver and got the disease when carpooling with Patient C. Patient H further transmitted the Delta-SARS-CoV-2 to his family (Patients I, J, and K), and Patients L, M, N, and O when carpooling or sharing food or drink. Both Patients P and Q caught the Delta-SARS-CoV-2 when carpooling, sharing food, or sharing infuse tea with Patients L, M, N, and O. The genomic sequences of SARS-CoV-2 from Patients J and N were not examined or determined due to high values of cycle threshold (Ct). Patient H was the first patient of record infected by Delta-SARS-CoV-2 in Taiwan. Based on tracing the contact history of Patient H, Patients A and B, who returned from Peru, were finally recognized as the original sources.

**Figure 2:**
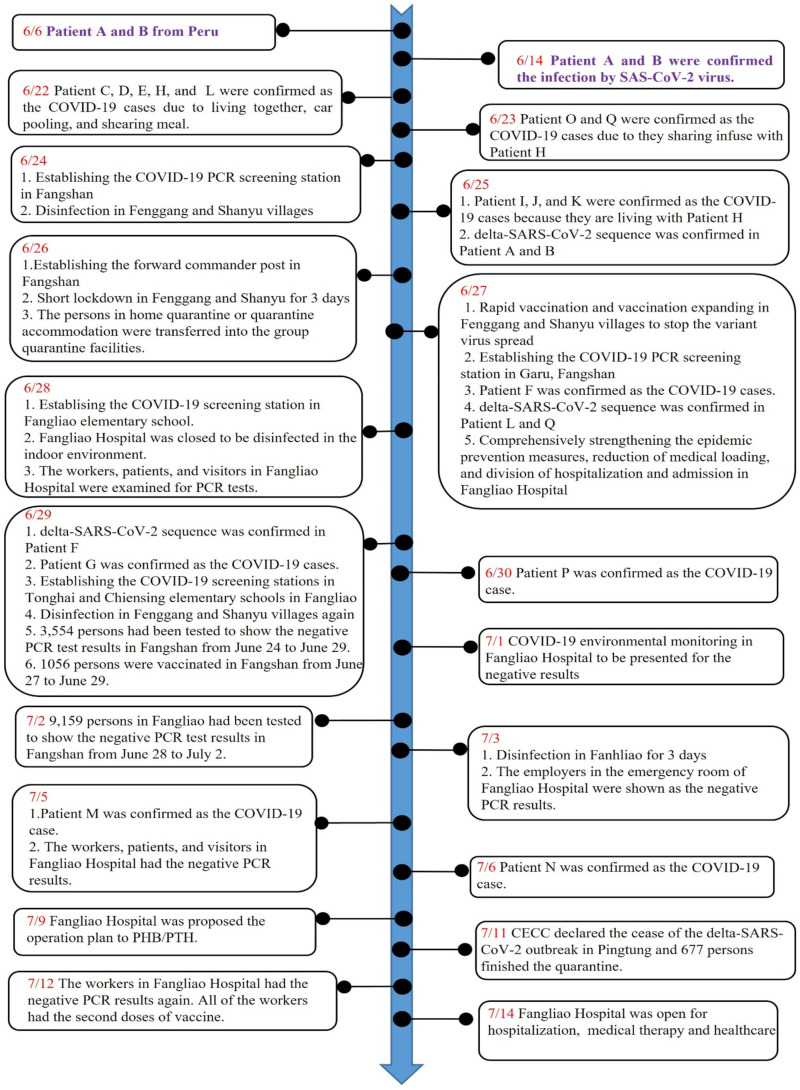
The timetable of rapid control of Delta-SARS-CoV-2 community spread.

The time course of fighting the Delta-SARS-CoV-2 by PHB/PTH is shown in Figure 2. After the originally infectious source was found on 14 June, 2021, PHB/PTH further found that the infectious transmission line between FS and FL may have been at the waiting rooms in the outpatient department of an FL Hospital. A screening station was then set up in FS on 24 June, and the FG and SY villages were disinfected. PHB/PTH established the forward commander post in SY village for assistance and coordination on 26 June. Immediately after confirmation of the causative Delta-SARS-CoV-2 through genome sequencing, PHB/PTH quickly expanded the isolation of 667 high-risk people and they were moved to centralized group quarantine facilities. Due to cluster infections in the two villages of FS Township, PHB/PTH not only rapidly performed short, local lockdowns in FG and SY villages, but also provided the food, water, and epidemic prevention materials for the people living in these two villages. In order to find more cases of community transmission, PHB/PTH had set up five screening stations to investigate the Delta-SARS-CoV-2. The real-time PCR analysis using SARS-CoV-2 specific primers showed 14,985 people with negative results. To avoid the transmission of Delta-SARS-CoV-2 in the FL Hospital due to Patients F and G living in FL who were infected in this hospital, the indoor environment was disinfected and three times of COVID-19 antigen screening was performed for the staff in the hospital. Fortunately, all of healthcare workers in the FL Hospital had negative results, and 42 points of the screening tests for COVID-19 antigen in the hospital environment also had negative results. One thousand and fifty-six people who lived in FG and SY villages were vaccinated with the AZ vaccine for rapid build-up of the immune system. After that, the FL Hospital was closed for 16 days and reopened on 14 July.

After several stages for control cluster infection in the community, home quarantine personnel were all discharged after 14 days of centralized quarantine, except for two cases that were infected with COVID-19 and were sent to the negative pressure of the centralized quarantine station ward for treatment. Unfortunately, one confirmed diagnosed COVID-19 case developed severe O_2_ unsaturation condition and passed away after 23 days (27 June to 21 July) of treatment. Other patients who were diagnosed with COVID-19 infection were discharged from the hospital after prompt treatment. Since 6 July, there have been no new cases of COVID-19 infection within the community of Pingtung County.

**Figure 3:**
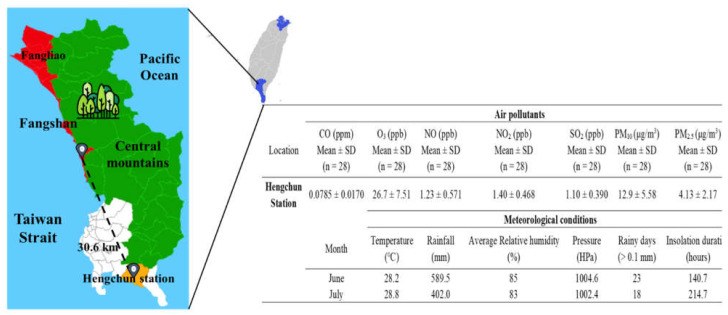
The outdoor air quality between 14 June and 11 July and the meteorological conditions of June and July in Hengchun station. Fangshan township and Fangliao township, red; Hengchun township, white; the downtown of Hengchun township, orange; Central mountains, green.

Severe air pollution is positively correlated with increased confirmed cases and mortality of COVID-19. The meteorological conditions are also associated with SARS-CoV-2 transmission. For the geography, FG and SY villages are rural areas between the seashore and high mountains, located in FS Township of Pingtung County (Figure 3). There are no air quality monitoring sites or a meteorological station in the two townships of FS and FL. The closest stations, located in Hengchun (Hengchun meteorological station, which is the most southern station in Taiwan) are 20–40 km from the pandemic areas. The air pollutants of CO, O_3_, NO, NO_2_, SO_2_, PM_10_, and PM_2.5_ were 0.0758 ± 0.0170 ppm, 26.7 ± 7.51 ppb, 1.23 ± 0.571 ppb, 1.40 ± 0.468 ppb, 1.10 ± 0.390 ppb, 12.9 ± 5.58 μg/m^3^, and 4.13 ± 2.17 μg/m^3^, respectively (Figure 3). All of the air pollutants in Hengchun station met the Taiwanese EPA standards and WHO guidelines. Compared with these levels in 2019, levels of NO_2_, SO_2_, PM_10_, and PM_2.5_ were lower but insignificantly different in 2021 (Supplementary Table S1). In addition, this study also compared the air quality in different regions of Taiwan. Geographically, Taipei City and New Taipei City are located in northern Taiwan, whereas Hengchun is a township located on the southern tip in Pingtung County of Taiwan (Figure S1). Meteorological station data analysis found that the air quality in Hengchun station was significantly lower than that in those stations located at Taipei and New Taipei City from 2019 to 2021 (Tables S2 and S3). For the meteorology, there were no significant differences in the meteorological conditions between Hengchun, Taipei City, and New Taipei City during the Delta-SARS-CoV-2 outbreak in Taiwan (Figure 3 and Table S4).

## 4. Discussion

Figure 1 and Figure 2 described the Delta-variant Pingtung outbreak in detail. The severe 14 day mandatory quarantine for the visitors or citizens arriving from abroad was carried out in Taiwan since the outbreak of Alpha-SARS-CoV-2 in Taipei City in the of middle May in 2021. CECC barred most non-citizens from entering Taiwan and put most persons in hotel quarantine, but allowed the eligible residents to serve home quarantine, like the woman and her grandson returning from Peru after the Alpha-variant outbreak. How could Patient D get the Delta-variant infection? The reasonable explanation is as follows. The first identified patient (Patient H), who was a taxi driver, was believed to have caught the Delta-SARS-CoV-2 from his passenger (Patient C) who caught the virus from his cohabitant (Patient D) infected by the next-door relative (Patient B) returning from Peru while Patient B put the garbage at the doorway to have a fleeting chat with Patient D. Although Patient B claimed to talk to Patient D with social distancing for a very short period, there is no evidence and it is still unknown why Patient D caught the Delta-variant from Patient B (indirect contact or airborne transmission). After the outbreak of Delta-variant in Taiwan, CECC declared 14 + 7 day quarantine in hotels or group quarantine facilities. The following was suspected for overlapping transmission from Patient D to Patient F: (1) Patient D and Patient F overlapped in the same place for hours and (2) the viral sequences of Patient F were notably associated with those of Patient D, indicating that the Delta-variant in Patient F was possibly originated from that in Patient D.

Rapid control of Delta-SARS-CoV-2 might be connected to the experience of a SARS outbreak in 2003. During 2003, SARS was efficiently transmitted between patients, healthcare workers, and visitors in hospitals. In Taiwan, SARS caused 346 definite cases and 73 deaths, which caused a great panic within the overall Taiwan population in the first several weeks. The Quarantine of Ho Ping Hospital also increased the risk of cross-infection among patients, visitors, clinicians, and other employees in the hospital [18]. The painful memories of the SARS outbreak in 2003 helped the Taiwanese fight SARS-CoV-2. Ever since the emergence of Alpha-SARS-CoV-2, the Ministry of Health and Welfare (MOHW) of Taiwan used several measures, such as imposing a 14-day quarantine requirement for all arrivals, isolated suspected cases, prohibiting the export of protective masks, avoiding unnecessary hospital visits, preparing enough negative pressure isolation wards and beds at hospitals across Taiwan, establishing an efficient virus diagnosis by using new technology and data, using proactive case detection, announcing incentive plans for frontline health care workers and business, closing entertainment venues and some educational institutions, providing transparent information, asking people to maintain social distancing and use protective masks in public areas, and performing temperature checks at every entrance of shops and buildings [19]. The Taiwanese government has also used a well-established National Health Insurance System to minimize the infringement of personal privacy and maximize the benefit of public health security, prevent panic hoarding through a mask rationing system, and provide medical information in epidemic investigation [20]; furthermore, the government set up data linkage systems and information-driven data linkage systems: these important tools help us better deal with the SARS-CoV-2 situation. The experience of SARS encourages people (especially health care workers) to proactively wear full protective equipment (including eye protection, protective suits, aprons, gloves, head and foot coverings, and respirators or masks) to protect themselves from SARS-CoV-2 and reduce the risk of SARS-CoV-2 outbreaks.

Since Delta-SARS-CoV-2 was first detected in India in December, 2020, it has contributed to 99% of the COVID-19 cases and accelerated the increase in hospitalization [7]. The Delta-SARS-CoV-2 had such an impact is because it grows extremely rapidly within human cells, has a higher viral load (up to 1000 times) than the original strain, has a higher rate of transmission and secondary attack rate, and has a potential reduction in neutralization by some monoclonal antibody treatments [4,5,6,21]. Moreover, the COVID-19 vaccines seem to be less effective against the Delta-SARS-CoV-2 [8], which indicates that vaccinated people with symptomatic breakthrough infections may be sources of SARS-CoV-2 transmission to others after infection with the Delta-SARS-CoV-2. In Taiwan, Delta-SARS-CoV-2 was identified in Pingtung County on June 14, 2021 (Figure 2). The strategies such as non-pharmaceutical intervention (NPI) and pharmaceutical intervention (PI) (including epidemic investigation, rapid gene sequencing, rapid expanding isolation measures, expanding screening of the COVID-19 antigen for people who lived in regional villages, short regional lockdown and travel restrictions, and rapid COVID-19 vaccination) and the environmental conditions of Pingtung County helped the Taiwanese government control and prevent Delta-SARS-CoV-2 spread. Finally, there were no new cases of COVID-19 infection within the community since July 6. Therefore, preventing new infections by early identification and control dissemination are essential for limiting COVID-19 impact.

Recently, the new SARS-CoV-2 variant, B.1.1.529 (Omicron), was confirmed from a specimen collected on 9 November, 2021 [3]. Up to the present, people infected with Omicron have been detected in at least 19 countries [22]. Although South African doctor, Angelique Coetzee, observed that the symptoms of Omicron seem ‘mild’ so far, her initial observations were only based on a very small number of cases [23]. Furthermore, the WHO indicated the Omicron had a high number of mutations, which may have immune escape potential and higher transmissibility. As compared to other variants, the Omicron also has an increased risk of reinfection [24]. The WHO also indicated that assessments of transmissibility, severity of infection (including symptoms), performance of vaccines and diagnostic tests, and effectiveness of treatments still need further study to better understand Omicron [24]. However, our successful experience in rapid control of Delta-SARS-CoV-2 may be useful for limiting Omicron impact.

The environmental factors, including air quality, meteorological conditions, and geographical locations, might be associated with the SARS-CoV-2 outbreak. For example, the air pollutants, such as PM_2.5,_ PM_10_, and NO_2_, and the strong wind speed were obviously and positively correlated with acceleration of the COVID-19 spread (e.g., the increased morbidity and mortality of COVID-19 cases); inversely, the negative association of high temperature and relative humidity and solar radiation with the coronavirus spread were observed [25,26,27,28]. The positive association of the air pollutants with SARS-CoV-2 infection mortality were mainly due to combinations of COVID-19 and air pollution related comorbidities in several reports [25,29,30,31]. The implementation of lockdown or restriction was demonstrated to improve the air quality in the regions during the duration of COVID-19 [25,32,33,34]. In the present study (Figure 3 and Tables S1–S3), compared with the corresponding periods in 2019 and 2020, the decline of PM_2.5_, PM_10_, and NO_2_ in 2021 were probably associated with restricted activities in the domestic areas and lockdown implementation after the Pingtung outbreak. In Figure 3, Figure S1, and Table S4, local meteorological data in the Delta-SARS-CoV-2 pandemic areas in the tropical zone were similar to those in Taipei and New Taipei City, which are located at the subtropical zone in the summer season in Taiwan. Compared with the continuous Alpha-variant of SARS-CoV-2 spread and outbreak in Taipei and New Taipei City from May to August, the quick end of the Delta-SARS-CoV-2 spread in Pingtung within 19 days (from 22 June to 10 July) was probably associated with low levels of air pollutants, particularly for PM_2.5_ and the obvious direction of land-sea breeze, low population density, and local geography (e.g., rural area on the coast near the mountains). The environmental factors might be a minor contribution to the rapid control of the Delta-variant Pingtung outbreak.

Previous studies revealed the hosts could be infected by SARS-CoV-2 through airborne transmission [8,10,11,12]. Delta-SARS-CoV-2 could possibly be transmitted via airborne transmission or environmental contamination. Several cases did not have close contact. Patient A took out the garbage to have a fleeting encounter with Patient D outdoors. Patients F and G who are a couple living in FL overlapped with Patient D in the same hospital (FL Hospital) waiting room. Based on the Mikszewski’s study [12], the Delta-SARS-CoV-2 is more easily transmitted from the victim to the host than the wild-type virus. Currently, the Omicron variant is rapidly spreading over the world. It is worth noting how fast the transmission of Omicron is via airborne transmission.

The rapid control of the Delta-SARS-CoV-2 outbreak in Pingtung was based on the quick response of the Pingtung County government with several key elements: speed, decisiveness, and overwhelming community cooperation with restrictions and contact tracing [35]. Strictly direct intervention included detailed contact tracing or epidemiologic investigation, rapid determination of Delta-SARS-CoV-2 genomic sequence, rapid expanded isolation of high-risk cases or close contacts (667 residents), and expanded PCR tests of SARS-CoV-2 for 14,985 residents. The PHB/PTH also used indirect interventions, like short lockdowns for 3 days, mandatory face masks, and rapid vaccination of 1056 residents. The direct and indirect interventions by CDC and PHB/PTH accelerated rapid control of Delta-SARS-CoV-2 spread. The inadvertently indirect environmental factors, such as low emission of air pollution, low-density population, tropical weather in summer season, and the rural areas between the coast and the mountains, might be minor factors that helped to stop the Pingtung Delta-SARS-CoV-2 outbreak within 1 month. The previous study reported that indirectly preventive measures like mandatory face masks, the implementation of lockdown leading to restriction of human activities and improvement of air quality, and vaccination is probably limited for the spread of the different SARS-CoV-2 variants [36]. No new COVID-19 cases including Omicron were found in Pingtung after the end of the Pingtung Delta-variant outbreak (5 July, 2021) until the present (27 January, 2022). Inversely, more than 10,000 COVID-19 cases were recorded in northern Taiwan, particularly in Taipei and New Taipei City between Mid-May and late July after the Alpha-SARS-CoV-2 outbreak. A few new cases of COVID-19 are continuously announced by CECC since CECC declared the cease of the outbreak in Taipei and New Taipei City (15 August, 2021). The most sufficient and centralized healthcare systems are supported in Taipei and New Taipei City in Taiwan. The rural community of FS and FL have insufficiently resourced health systems, 20% of the residents are over 65 years old, and no one was vaccinated before the Pingtung outbreak. Compared with the continuous spread of Alpha-SARS-CoV-2 in Taipei and New Taipei City, which are sufficiently resourced health systems, the effective intervention from PHB/PTH and indirect environmental factors might be a useful protocol to eliminate the Delta-SARS-CoV-2 spread. Based on no vaccination and insufficiently resourced health systems in the pandemic areas before the outbreak in Pingtung, the successful experience in rapid control of Delta-SARS-CoV-2 could provide a successful formula to control the spread of Delta-SARS-CoV-2 or a new variant like Omicron in unvaccinated or insufficiently vaccinated regions. This successful experience might help WHO make globally new guidelines for the rapid control of Delta-SARS-CoV-2 spread, a new variant (i.e., Omicron), or future unknown variants of SASR-CoV-2. especially for areas with insufficient healthcare systems or vaccination shortages in underdeveloped countries in Africa, Southeast Asia, or South America.

The limitations of the present study were the following: first, this study was not designed for control of the Delta-variant SARS-CoV-2 spread, and it was a real case for the control of the infectious disease. It was difficult to design the case-control study for examining outcomes with and without control measures due to the Delta-variant’s high levels of infection and mortality. Second, the community transmission of Delta-variant SARS-CoV-2 viruses was a sudden outbreak, and implementation of the control measures was chaos in the beginning. Although the implemented control measures, including the detailed epidemiological investigation, quarantine, PCR examination, determination genomic sequencing, vaccination, and lockdown, which were simultaneously carried out, had effective reduction and control of the outbreak, the main impact on the infectious control of Delta-variant among these measures could not be determined due to lack of evidence. Third, the environmental factors including air quality, meteorological conditions, and geographical location were gathered from official documents. The data of air quality and meteorology were recorded in Hengchun station, which is the most southern station in Taiwan, 20–40 km away from the pandemic areas. Fourth, the pandemic areas are located at the rural areas in the tropical zone and June and July is a hot and wet season in the pandemic areas.

## 5. Conclusions

Pingtung rapid control of Delta-SARS-CoV-2 outbreak was probably due to implementation of the successful formula including the mandatorily direct and indirect intervention and inadvertently indirect environmental effects. This is the first case to success the rapid control of Delta-variant of SARS-CoV-2 spread in the world. The Pingtung outbreak was probably due to the implementation of the successful formula which may potentially be applied on or referenced by the unvaccinated or insufficiently vaccinated regions, particularly for the countries in Africa, South America, Southeast Asia, and the Far East Regions. Furthermore, the successful experience in rapid control of Delta-SARS-CoV-2 may also be useful for new variant (e.g., Omicron) control.

## Data Availability

The original contributions presented in the study are included in the article.

## Supplementary Materials

The following are available online at https://www.mdpi.com/article/10.3390/ijerph19031421/s1, Figure S1: The relative position of Taipei, New Taipei, and Pingtung in Taiwan. The pandemic Delta-SARS-CoV-2 began in FS and FL in Pingtung. Table S1: Average of air-quality at Hengchun stations from 14 June to 11 July between 2019 and 2021 in Pingtung. Table S2: Average of air-quality at Shilin, Wanhua, and Songshan stations from 14 June to 11 July between 2019 and 2021 in Taipei. Table S3: Average of air-quality at Yonghe, Banqiao, and Xinzhung stations from 14 June to 11 July between 2019 and 2021 in New Taipei. Table S4: Measurement of the meteorological stations comparing the COVID-19 pandemic for 2021–2019.
