# Supplementary material for: Rapid Control of a SARS-CoV-2 B.1.617.2 (Delta) Variant COVID-19 Community Outbreak: The Successful Experience in Pingtung County of Taiwan
Source: Int J Environ Res Public Health. 2022 Jan 27;19(3):1421. doi: 10.3390/ijerph19031421 (PMC8834902; doi:10.3390/ijerph19031421)
Supplement: Supplementary file 1 [file ijerph-19-01421-s001.zip › ijerph-1546746-supplementary.pdf]

Supplementary Materials

Rapid Control of a SARS-CoV-2 B.1.617.2 (Delta) variant COVID-19 Community Outbreak: the Successful Experience in Pingtung County of Taiwan

Cherng-Gueih Shy [1,2,+], Jian-He Lu [3], Hui-Chen Lin [4], Min-Nan Hung [4], Hsiu-Chun Chang [1], Meng-Lun Lu [1], How-Ran Chao [3,5,6,7,*,+], Yao-Shen Chen [8,9,+] and Pi-Sheng Wang [10]

[1] Public Health Bureau, Pingtung County Government, Pingtung, Pingtung County 900, Taiwan; pthh7362396@mail.ptshb.gov.tw (C.-G. S); pth0905981587@mail.ptshb.gov.tw (H.-C. C); pthmen-glun@mail.ptshb.gov.tw (M.-L. L)

[2] Department of Radiology, Pingtung Christian Hospital, Pingtung, Pingtung County 900, Taiwan

[3] Emerging Compounds Research Center, General Research Service Center, National Pingtung University of Science and Technology, Neipu, Pingtung County 912, Taiwan; toddherpuma@mail.npust.edu.tw

[4] Kaohsiung-Pingtung Regional Center, Taiwan Centers for Disease Control, Ministry of Health and Welfare, Executive Yuan, Taipei City 10050, Taiwan; lhuichen@cdc.gov.tw (H.-C. L); mnhung@cdc.gov.tw (M.-N. H)

[5] Department of Environmental Science and Engineering, College of Engineering, National Pingtung University of Science and Technology, Neipu, Pingtung County 912, Taiwan; hrchao@mail.npust.edu.tw

[6] Institute of Food Safety Management, College of Agriculture, National Pingtung University of Science and Technology, Neipu, Pingtung County 912, Taiwan

[7] School of Dentistry, College of Dental Medicine, Kaohsiung Medical University, Kaohsiung City 807, Taiwan.

[8] Department of Administration, Kaohsiung Veterans General Hospital, Kaohsiung, Taiwan; yschen@vghks.gov.tw

[9] School of Medicine, National Yang Ming Chiao Tung University, Taipei, Taiwan

[10] Hospital and Social Welfare Organizations Administration Commission, Ministry of Health and Welfare , Chief Executive Officer, Nangang, Taipei city 11558, Taiwan; hmpswang@mohw.gov.tw

\+ These authors contributed equally.

* Correspondence: hrchao@mail.npust.edu.tw; Tel.: 886-87703202 (ext. 7517); Fax: +886-87740256

The environmental factors in relation to the delta-SARS-CoV-2 spread in Taiwan

A review article reported by Dr. Sirvastava was conducted that the increased $PM_{2.5}$, PM_{10}, and NO_2 was positively associated with the increased morbidity and mortality of COVID-19 cases and the decrease in the confirmed COVID-19 cases was related with the rising temperature and solar radiation from the several published data [25]. The implementation of lockdown was demonstrated to improve the air quality in the regions during the duration of COVID-19 [25]. In our previous study [26], the reduction of $PM_{2.5}$ by 10.6% in northern Taiwan and 3.70% in southern Taiwan between January and March from 2019 to 2020 was observed due to the decreased $PM_{2.5}$ emissions from restricted activities in the local areas and the long-range transport of reduced $PM_{2.5}$ through winter monsoon from the mainland China during the COVID-19 issue. After the restricted activities or lockdown in Taiwan since 2020, the air quality was improved. According to Table 1 and Table S1 and S2, the decline of $PM_{2.5}$, PM_{10}, and NO_2 in 2021 was probably associated with restricted activities in the domestic areas and lockdown implementation after the Pingtung outbreak. The air pollution like $PM_{2.5}$ and NO_2 is proved as a main contribution to trigger the COVID-19 spread and lethality based on the evidence of airborne transmission [27]. The delta variant of SARS-CoV-2 is infected faster than the wild-type SARS-CoV-2 via airborne transmission [28]. The Pingtung outbreak of delta variant was rapidly controlled within 3 weeks and the alpha variant outbreak in Taipei and New Taipei City was continuously transmitted between May and August in 2021. Whether can we use the air quality to explain the rapid control of delta variant? The answer might be not the main contributor, but good air quality possibly provides the positively minor effect. The air pollutants of CO, NO, NO_2, SO_2, PM_{10}, and $PM_{2.5}$ in Hengchun (the rural areas) were significantly lower than those in Taipei or New Taipei City (the urban areas), indicating that the more human and economic activities are produced the more air pollutants. The air pollution was significantly decreased during implementation of lockdown compared with those in the same areas without lockdown [29, 30]. According to the previous studies[25, 31-33], the positive associations of the air pollutants with SARS-CoV-2 infection mortality was mainly due to combination of COVID-19 and air pollution related comorbidities. The British study indicated the NO_2 and $PM_{2.5}$ increase of every 1.00 $\mu g/m^3$ to enhance 0.5 and 1.4% increase in COVID-19 mortality risk, respectively, by Bayesian hierarchical models after the confounders of meteorological (e.g., temperature and relative humidity), spatial (e.g., urban and rural areas), healthcare (e.g., occupational exposure and intensive care beds), and health (e.g., obesity and smoking) confounders were adjusted [32]. Local meteorological data in the delta-SARS-CoV-2 pandemic areas were similar to those in Taipei and New Taipei City due to the summer season in Taiwan. Local meteorology might play an important role in the SARS-CoV-2 spread and COVID-19 mortality, such as the negative associations of high temperature and relative humidity and solar radiation with the corona virus spread and inversely, acceleration of the COVID-19 spread by the strong wind speed [25, 34, 35]. Compared with the continuous alpha variant of SARS-CoV-2 spread and outbreak in Taipei and New Taipei City from May to August, the quick cease of the delta-SARS-CoV2 spread in Pingtung within 19 days (from June 22 to July 10) was probably associated with low levels of air pollutants, particularly for $PM_{2.5}$ and the obvious direction of land-see breeze, low population density, and local geography (e.g., rural area on the coast near the mountains).

References

25. Srivastava, A., COVID-19 and air pollution and meteorology-an intricate relationship: A review. *Chemosphere* **2021,** 263.

26. Yu, T. Y.; Chao, H. R.; Tsai, M. H.; Lin, C. C.; Lu, I. C.; Chang, W. H.; Chen, C. C.; Wang, L. J.; Lin, E. T.; Chang, C. T.; Chen, C. N.; Kao, C. C.; Mansor, W. N. W.; Yu, K. L. J., Big Data Analysis for Effects of the COVID-19 Outbreak on Ambient PM2.5 in Areas that Were Not Locked Down. *Aerosol Air Qual Res* **2021,** 21, (8).

27. Copat, C.; Cristaldi, A.; Fiore, M.; Grasso, A.; Zuccarello, P.; Signorelli, S. S.; Conti, G. O.; Ferrante, M., The role of air pollution (PM and NO2) in COVID-19 spread and lethality: A systematic review. *Environ Res* **2020,** 191, 110129.

28. Mikszewski, A.; Stabile, L.; Buonanno, G.; Morawska, L., Increased close proximity airborne transmission of the SARS-CoV-2 Delta variant. *Sci Total Environ* **2021**, 151499.

29. Kumari, S.; Lakhani, A.; Kumari, K. M., COVID-19 and Air Pollution in Indian Cities: World's Most Polluted Cities. *Aerosol Air Qual Res* **2020,** 20, (12), 2592-2603.
30. Zhu, S. H.; Wang, Q. Q.; Qiao, L. P.; Zhou, M.; Wang, S.; Lou, S. R.; Huang, D. D.; Wang, Q.; Jing, S. G.; Wang, H. L.; Chen, C. H.; Huang, C.; Yu, J. Z., Tracer-based characterization of source variations of PM2.5 and organic carbon in Shanghai influenced by the COVID-19 lockdown. *Faraday Discuss* **2021,** 226, 112-137.
31. Borro, M.; Di Girolamo, P.; Gentile, G.; De Luca, O.; Preissner, R.; Marcolongo, A.; Ferracuti, S.; Simmaco, M., Evidence-Based Considerations Exploring Relations between SARS-CoV-2 Pandemic and Air Pollution: Involvement of PM2.5-Mediated Up-Regulation of the Viral Receptor ACE-2. *Int J Env Res Pub He* **2020,** 17, (15).
32. Konstantinoudis, G.; Padellini, T.; Bennett, J.; Davies, B.; Ezzati, M.; Blangiardo, M., Long-term exposure to air-pollution and COVID-19 mortality in England: A hierarchical spatial analysis. *Environment International* **2021,** 146.
33. Sharma, A. K.; Balyan, P., Air Pollution and COVID-19: Is the Connect Worth Its Weight? *Indian J Public Hlth* **2020,** 64, 132-134.
34. Wei, J. T.; Liu, Y. X.; Zhu, Y. C.; Qian, J.; Ye, R. Z.; Li, C. Y.; Ji, X. K.; Li, H. K.; Qi, C.; Wang, Y.; Yang, F.; Zhou, Y. H.; Yan, R.; Cui, X. M.; Liu, Y. L.; Jia, N.; Li, S. X.; Li, X. J.; Xue, F. Z.; Zhao, L.; Cao, W. C.; CLEC, C. E. C., Impacts of transportation and meteorological factors on the transmission of COVID-19. *Int J Hyg Envir Heal* **2020,** 230.
35. Adhikari, A.; Yin, J. J., Short-Term Effects of Ambient Ozone, PM2.5, and Meteorological Factors on COVID-19 Confirmed Cases and Deaths in Queens, New York. *Int J Env Res Pub He* **2020,** 17, (11).

Table S1. Average of air-quality at Hengchun stations form June 14th to July 11th between 2019 and 2021 in Pingtung.

		Air pollutants						
Location	Years	CO (ppm) Mean ± SD (min – Max)	O_3 (ppb) Mean ± SD (min – Max)	NO (ppb) Mean ± SD (min – Max)	NO_2 (ppb) Mean ± SD (min – Max)	SO_2 (ppb) Mean ± SD (min – Max)	PM_{10} (μg/m^3) Mean ± SD (min – Max)	$PM_{2.5}$ (μg/m^3) Mean ± SD (min – Max)
Pingtung								
Hengchun	2021	0.0785 ± 0.0170	26.7 ± 7.51	1.23 ± 0.571	1.40 ± 0.468	1.10 ± 0.390	12.9 ± 5.58	4.13 ± 2.17
		(0.047 – 0.108)	(14.4 – 40.8)	(0.312 – 2.88)	(0.335 – 2.45)	(0.267 – 1.92)	(6.17 – 27.9)	(1.46 – 10.2)
	2020	0.0516 ± 0.000408	21.9 ± 2.88	1.20 ± 0.49	0.903 ± 0.43	0.700 ± 0.258	7.40 ± 2.58	4.16 ± 0.979
		(0.05 – 0.07)	(15.2 – 30.6)	(0.40 – 2.43)	(0.34 – 2.34)	(0.48 – 1.42)	(4.58 – 14.9)	(2.54 – 6.39)
	2019	0.0517 ± 0.0265	25.1 ± 3.04	0.852 ± 0.276	1.42 ± 0.32	1.35 ± 0.439	13.0 ± 9.75	6.24 ± 3.09
		(0.05 – 0.1248)	(19 – 31.1)	(0.333 – 1.34)	(0 – 2.00)	(0.500 – 2.10)	(0 – 36.9)	(0 – 10.6)

Table S2. Average of air-quality at Shilin, Wanhua, and Songshan stations form June 14th to July 11th between 2019 and 2021 in Taipei.

		Air pollutants						
Location	Years	CO (ppm) Mean ± SD (min – Max)	O_3 (ppb) Mean ± SD (min – Max)	NO (ppb) Mean ± SD (min – Max)	NO_2 (ppb) Mean ± SD (min – Max)	SO_2 (ppb) Mean ± SD (min – Max)	PM_{10} (μg/m^3) Mean ± SD (min – Max)	$PM_{2.5}$ (μg/m^3) Mean ± SD (min – Max)
Taipei								
Shilin	2021	0.207 ± 0.052	26.7 ± 6.97	2.04 ± 0.890	8.81 ± 2.61	8.81 ± 2.61	21.6 ± 5.29	9.04 ± 2.40
		(0.089 – 0.34)	(2.20 – 35.1)	(0.180 – 3.89)	(4.17 – 15.3)	(1.23 – 4.15)	(9.88 – 29.6)	(3.67 – 14.7)
	2020	0.289 ± 0.0356	23.8 ± 4.11	2.88 ± 1.12	12.4 ± 1.63	2.33 ± 0.515	24.2 ± 2.97	10.1 ± 1.46
		(0.241 – 0.386)	(15.5 – 32.0)	(0.779 – 5.40)	(9.43 – 15.4)	(1.44 – 3.11)	(16.2 – 29.6)	(7.33 – 13.7)
	2019	0.315 ± 0.0639	28.7 ± 10.6	2.98 ± 1.49	12.5 ± 2.35	2.12 ± 0.431	19.2 ± 10.2	12.1 ± 2.90
		(0.216 – 0.454)	(16.2 – 62.1)	(0.34 – 6.38)	(7.36 – 17.0)	(1.16 – 2.94)	(3.33 – 36.4)	(5.71 – 17.7)
Wanhua	2021	0.274 ± 0.0670	25.4 ± 4.06	2.57 ± 1.01	10.8 ± 3.60	2.06 ± 0.598	19.6 ± 3.49	9.71 ± 2.49
		(0.164 – 0.468)	(16.7 – 36.2)	(0.81 – 4.93)	(5.25 – 20.5)	(1.17 – 3.20)	(12.2 – 25.3)	(4.80 – 15.2)
	2020	0.372 ± 0.0412	23.0 ± 3.71	4.19 ± 1.72	14.5 ± 2.21	1.75 ± 0.555	22.2 ± 1.65	9.13 ± 1.46
		(0.302 – 0.489)	(16.1 – 29.3)	(1.80 – 10.1)	(11.4 – 19.6)	(0.575 – 2.94)	(17.2 – 24.4)	(5.79 – 11.7)
	2019	0.437 ± 0.065	22.0 ± 9.9	4.70 ± 1.29	17.3 ± 3.18	1.84 ± 0.59	20.8 ± 7.0	10.0 ± 2.68
		(0.345 – 0.563)	(10.1 – 52.6)	(1.98 – 7.88)	(12.3 – 23.0)	(0.75 – 3.02)	(4.58 – 29.5)	(5.38 – 15.5)
Songshan	2021	0.272 ± 0.0576	25.7 ± 5.50	3.51 ± 1.90	11.2 ± 2.59	2.35 ± 0.599	19.4 ± 5.14	9.36 ± 2.87
		(0.147 – 0.372)	(14.1 – 38.3)	(0.800 – 7.64)	(4.82 – 16)	(0.929 – 3.37)	(10.2 – 28.7)	(3.50 – 16.6)
	2020	0.36 ± 0.0556	24.2 ± 4.42	6.15 ± 2.35	15.6 ± 2.64	2.09 ± 0.628	23.3 ± 1.97	11.2 ± 1.51
		(0.256 – 0.505)	(16.6 – 33.7)	(2.74 – 10.6)	(9.82 – 21.6)	(0.5 – 3.05)	(18.4 – 26)	(7.14 – 14.4)
	2019	0.378 ± 0.0644	24.4 ± 10.1	6.17 ± 3.09	16.2 ± 3.31	2.27 ± 0.63	19.4 ± 11.3	12 ± 2.54
		(0.234 – 0.492)	(11.8 – 58.6)	(1.64 – 16.2)	(9.52 – 23.6)	(0.35 – 3.27)	(0 – 37)	(6 – 16.9)

Table S3. Average of air-quality at Yonghe, Banqiao, and Xinzhung stations form June 14th to July 11th between 2019 and 2021 in New Taipei.

		Air pollutants						
Location	Years	CO (ppm) Mean ± SD (min – Max)	O_3 (ppb) Mean ± SD (min – Max)	NO (ppb) Mean ± SD (min – Max)	NO_2 (ppb) Mean ± SD (min – Max)	SO_2 (ppb) Mean ± SD (min – Max)	PM_{10} (μg/m^3) Mean ± SD (min – Max)	$PM_{2.5}$ (μg/m^3) Mean ± SD (min – Max)
New Taipei								
Yonghe	2021	0.440 ± 0.133	24.1 ± 4.08	5.53 ± 2.16	11.2 ± 3.81	1.91 ± 0.719	20.5 ± 3.92	9.87 ± 2.61
		(0.24 – 0.808)	(15.2 – 32.9)	(1.87 – 2.04)	(2.49 – 11.8)	(0.89 – 3.78)	(11.7 – 26.3)	(5.33 – 15.3)
	2020	0.577 ± 0.0798	22.6 ± 3.24	8.08 ± 1.97	14 ± 2.19	1.56 ± 0.617	19 ± 1.49	10.1 ± 1.29
		(0.744 – 0.453)	(28.5 – 17.4)	(12.2 – 4.97)	(19.2 – 9.52)	(2.85 – 0.932)	(23.6 – 16.5)	(12.7 – 6.33)
	2019	0.67 ± 0.131	23 ± 10.1	9.21 ± 2.83	16.9 ± 3.6	1.96 ± 0.658	23 ± 9.77	10.4 ± 5.28
		(0.432 – 0.892)	(11.6 – 55.6)	(3.49 – 14.9)	(10.8 – 26.1)	(0.704 – 2.92)	(0 – 36)	(2.71 – 23.1)
Banqiao	2021	0.234 ± 0.0663	23.3 ± 3.69	1.67 ± 0.459	9.83 ± 2.88	2.72 ± 0.832	20.1 ± 3.93	9.01 ± 2.61
		(0.153 – 0.400)	(15.0 – 30.7)	(1.83 – 1.98)	(0.833 – 2.62)	(0.89 – 3.89)	(11.7 – 27.8)	(4.96 – 15.1)
	2020	0.328 ± 0.0622	22.6 ± 3.21	3.24 ± 1.28	13.8±2.09	2.69 ± 0.910	21.4 ± 2.04	8.57 ± 1.41
		(0.259 – 0.510)	(16.7 – 28.2)	(1.38 – 6.5)	(10.7 – 18.2)	(0.500 – 5.70)	(17.0 – 25.1)	(5.96 – 12.1)
	2019	0.421 ± 0.107	23.9 ± 8.89	3.71 ± 1.48	16.8 ± 3.80	2.76 ± 0.89	23.8 ± 6.5	13.5 ± 2.86
		(0.242 – 0.658)	(13.9 – 54.8)	(1.42 – 7.35)	(8.96 – 24.7)	(1.36 – 4.85)	(14.4 – 34.4)	(8.46 – 19.2)
Xinzhuang	2021	0.254 ± 0.0677	26.0 ± 3.83	1.86 ± 0.621	9.23 ± 2.78	3.22 ± 0.845	18.6 ± 3.76	10.8 ± 2.74
		(0.143 – 0.392)	(18.9 – 36.4)	(1.00 – 3.19)	(4.93 – 14.6)	(1.72 – 4.74)	(9.40 – 25.2)	(5.83 – 15.8)
	2020	0.323 ± 0.054	24.0 ± 3.86	3.29 ± 1.25	12.7 ± 2.01	3.46 ± 0.98	24.4 ± 2.6	10.1 ± 1.80
		(0.243 – 0.460)	(15.1 – 31.5)	(1.38 – 5.97)	(8.49 – 17.6)	(1.38 – 5.17)	(20.0 – 30.7)	(6.92 – 15.6)
	2019	0.415 ± 0.111	25.9 ± 11.3	4.63 ± 2.54	15.7 ± 3.94	2.98 ± 0.83	16.7 ± 9.75	11.3 ± 3.10
		(0.250 – 0.607)	(8.20 – 55.9)	(1.90 – 11.84)	(8.51 – 22.1)	(1.94 – 4.36)	(0 – 36.5)	(5.54 – 17.3)

Table S4. Measurement of the meteorological stations compared the COVID-19 pandemic among the 2021-2019.

Location	Years	Temperature (℃)	Rainfall (mm)	Average Relative humidity (%)	Pressure (HPa)	Rainy days (> 0.1 mm)	Insolation duration (Hours)
June							
Hengchun	2021	28.2	589.5	85	1005	23	140.7
	2020	29.6	80.0	86	1006	10	260.2
	2019	28.9	215.5	84	1004	15	94.5
Taipei	2021	29.3	316.5	74	1003	10	152.4
	2020	30.5	117.9	68	1004	8	150.6
	2019	28.5	419.5	77	1003	16	91.4
Banqiao	2021	29.0	291.5	83	1005	13	179.6
	2020	30.0	108.3	71	1007	6	222.5
	2019	28.1	452.3	76	1005	16	127.9
July							
Hengchun	2021	28.8	402.0	83	1002	18	214.7
	2020	29.8	246.5	85	1006	13	284.2
	2019	28.9	587.5	86	1004	23	87.1
Taipei	2021	30.3	288.0	72	1001	13	217.2
	2020	30.9	133.5	67	1004	9	160.3
	2019	30.3	439.3	73	1002	16	145.5
Banqiao	2021	30.0	421.5	72	1003	15	221.5
	2020	30.4	256.0	70	1006	8	220.5
	2019	29.9	362.7	73	1004	16	195.3

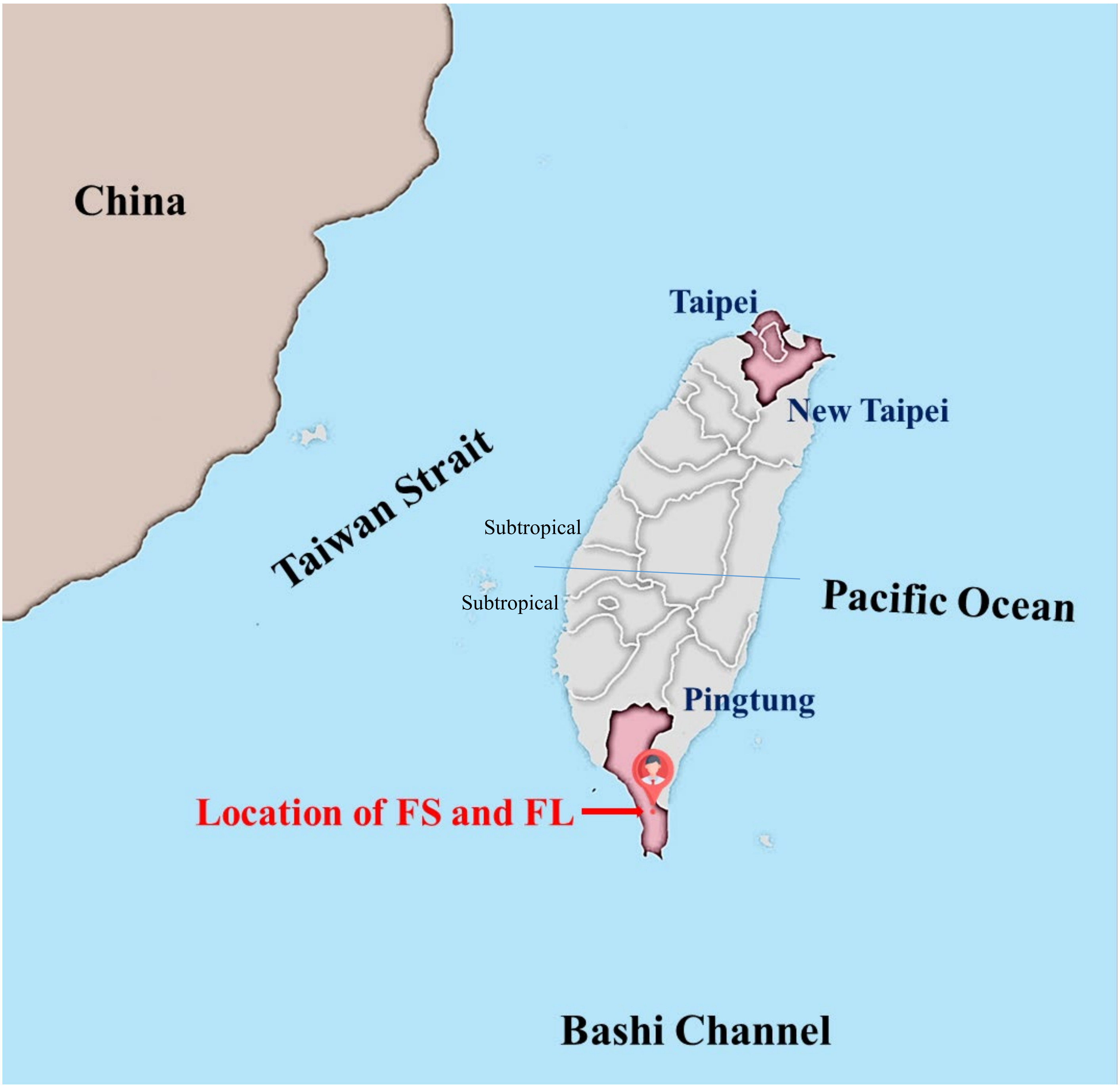

Figure S1. The relative position of Taipei, New Taipei, and Pingtung in Taiwan. The pandemic delta-SARS-CoV-2 began in FS and FL in Pingtung.
